# Whole body protein kinetics during hypocaloric and normocaloric feeding in critically ill patients

**DOI:** 10.1186/cc12837

**Published:** 2013-07-24

**Authors:** Agneta Berg, Olav Rooyackers, Bo-Michael Bellander, Jan Wernerman

**Affiliations:** 1 Department of Anesthesiology and Intensive Care Medicine, K32, Karolinska University Hospital Huddinge, Stockholm 14186, Sweden; 2 Department of Neurosurgery, Karolinska University Hospital Solna, at Karolinska Institutet, Stockholm, Sweden

**Keywords:** Protein synthesis, Protein degradation, Amino acid oxidation, Parenteral feeding

## Abstract

**Introduction:**

Optimal feeding of critically ill patients in the ICU is controversial. Existing guidelines rest on rather weak evidence. Whole body protein kinetics may be an attractive technique for assessing optimal protein intake. In this study, critically ill patients were investigated during hypocaloric and normocaloric IV nutrition.

**Methods:**

Neurosurgical patients on mechanical ventilation (n = 16) were studied during a 48-hour period. In random order 50% and 100% of measured energy expenditure was given as IV nutrition during 24 hours, corresponding to hypocaloric and normocaloric nutrition, respectively. At the end of each period, whole body protein turnover was measured using d5-phenylalanine and 13C-leucine tracers.

**Results:**

The phenylalanine tracer indicated that whole-body protein synthesis was lower during hypocaloric feeding, while whole-body protein degradation and amino acid oxidation were unaltered, which resulted in a more negative protein balance, namely −1.9 ± 2.1 versus −0.7 ± 1.3 mg phenylalanine/kg/h (*P* = 0.014). The leucine tracer indicated that whole body protein synthesis and degradation and amino acid oxidation were unaltered, but the protein balance was negative during hypocaloric feeding, namely −0.3 ± 0.5 versus 0.6 ± 0.5 mg leucine/kg/h (*P* < 0.001).

**Conclusion:**

In the patient group studied, hypocaloric feeding was associated with a more negative protein balance, but the amino acid oxidation was not different. The protein kinetics measurements and the study’s investigational protocol were useful for assessing the efficacy of nutrition support on protein metabolism in critically ill patients.

## Introduction

Feeding critically ill patients in the ICU is a controversial subject in many respects. Timing of feeding, caloric content, and protein content are all issues, and here, existing guidelines are based upon a low level of scientific evidence [1-3]. Why? Published studies are old, and often used obsolete techniques to evaluate the effects from feeding [4]. While physiology is unchanged, treatment (intensive care medicine) has changed, and this limits the validity of studies from the last millennium.

Nitrogen balance constitutes the classic measurement of protein intake effects. Limitations of the classic nitrogen balance technique are particularly pronounced in intensive care although most limitations are general. While control over intake and output may be sufficient, the steady-state criterion and adaption-to-intake criterion (both critical for interpretation of data) are rarely fulfilled. Meticulously performed nitrogen balance studies, however, form the foundation for current recommendations [5,6]. Reports from body composition studies are also part of the documentation behind current recommendations. Techniques based on impedance measurements are not reliable in critically ill patients (associated with rapid changes in body fluids). Neutrone activation is a very attractive technique to assess lean body mass [7] but low availability - and need for patient transfer for assessment - limit its usefulness. So far, no randomized prospective studies of protein intake in ICUs are available.

Whole body, protein kinetics measurements that use isotopically labeled amino acid should be the technique of choice for global assessments of protein metabolism in relation to protein intake. Protein synthesis and degradation can be assessed separately, and amino acid oxidation and whole-body protein balance can be calculated. The period during which a steady state is needed is relatively short, and measures may be repeated. Despite these obvious benefits - at least in theory - only a few studies of critically ill patients are available [8-10]. There are relatively more studies in burn patients [11,12], but the validity of these studies for trauma and sepsis patients in general ICUs is limited.

In this pilot study, we included a homogenous patient group in a neurosurgical ICU. To optimize study conditions, we limited inclusions to sedated, mechanically ventilated adult patients, who received only parenteral nutrition. This selection, of course, limits the generalizability of results. Our main objective was to compare hypocaloric and normocaloric feeding in terms of whole-body protein kinetics.

### Materials and methods

Patients admitted to the neurosurgical ICU at Karolinska Solna were studied in the 2009 to 2011 period. Inclusion criteria were (i) a diagnosis of traumatic brain injury or intracranial bleeding, (ii) sedated and on mechanical ventilation, and (iii) receiving parenteral nutrition. Exclusion criteria were (i) age <18 years, and (ii) absence of informed consent. The Ethics Committee of Karolinska *Institutet* approved the protocol and the patients' relatives gave informed consent after being informed verbally and in writing about the investigational procedure and possible risks.

**Figure 1:**
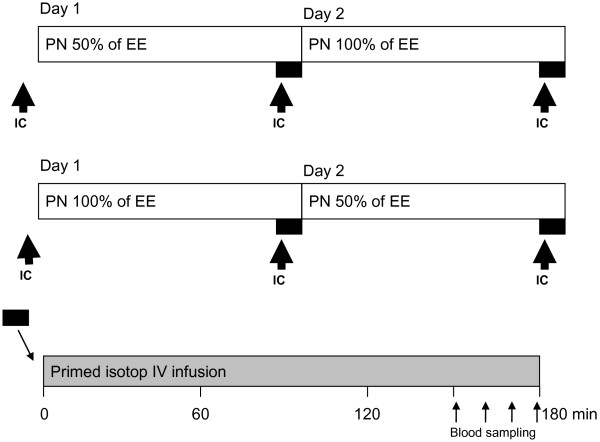
**A schematic illustration of the study protocol.** IC, indirect calorimetry; EE, energy expenditure; PN, parenteral nutrition; IV, intravenous.

The protocol included a 48-hour study period, starting with an energy expenditure measurement by indirect calorimetry during feeding (Deltatrac Metabolic Monitor, Datex-Ohmeda, Helsinki, Finland). Thereafter, patients were randomized to one of two alternatives, namely, to receive (i) 50% of the measured energy expenditure during 24 hours before receiving 100% during the consecutive 24 hours, or (ii) 100% of the measured energy expenditure during 24 hours followed by 50% of the measured energy expenditure during the next 24 hours. This protocol enabled patients to be their own controls, provided the two consecutive days were comparable (Figure 1).

Nutrition was delivered as an all-in-one formulation: Nutriflex Lipid Special (B Braun AG, Melsungen, Germany) contains a protein-to-energy ratio of 0.049 g/kcal. The intravenous (IV) nutrition infusion rate was set to give the amount of calories measured as energy expenditure by the indirect calorimetry. In cases when propofol was used for sedation, the IV nutrition infusion rate was adjusted accordingly. So, in practice this further lowered the administered protein-per-kg for most patients.

The whole-body protein turnover measurements were made using isotopically labeled leucine and phenylalanine in parallel. There were two study periods for each patient, at the end of each 24-hour period, with one of the two nutritional regimens.

On each occasion the patients received a primed continuous infusion of the labeled amino acids (Figure 1). The prime consisted of ring-^2^H_5_-phenylalanine (0.5 mg/kg), ring-^2^H_4_-tyrosine (0.15 mg/kg), 3,3-^2^H_2_-tyrosine (0.3 mg/kg), 1-^13^C-leucine (0.9 mg/kg) and ^13^C-sodium-bicarbonate (0.2 mg/kg). A continuous infusion of ring-^2^H_5_-phenylalanine (0.5 mg/kg/h), 3,3-^2^H_2_-tyrosine (0.3 mg/kg/h) and 1-^13^C-leucine (1.0 mg/kg/h) followed the prime.

All isotopes were obtained from Cambridge Isotopes Inc. (Cambridge, MA, USA). The hospital pharmacy prepared and tested sterile solutions for sterility and pyrogenity. Labeled amino acids were administered intravenously for 150 minutes before the sampling to obtain an isotopic steady state before the sampling period, which involved samples taken on 4 occasions during the last 30 minutes of the infusion. Blood samples were taken from an arterial line. Plasma was obtained and frozen at −80°C until analysis. Breath samples for ^13^CO_2_ measurements were taken from the DeltaTrac Metabolic Monitor during the 30-minute sampling periods. A Douglas bag was used, and the samples were transferred to vacutainers.

For analysis of the labeled amino acids, plasma was deproteinized using sulfosalisylic acid. The amino acids were cleaned using ion-exchange chromatography and dried using rotary evaporation. The amino acids were derivatized using N-(tert-butyldimethylsilyl)-N-methyltrifluoroacetamide (MTBSTFA). The amino acids were analyzed with an Agilent N5973 gas chromatography and mass spectrometer (GCMS; Agilent, Kista, Sweden) for m/z 336 and 341 for phenylalanine, 302 and 303 for leucine, and 466, 468, and 470 for tyrosine.

Keto-isocaproic acid (KIC) enrichment in plasma was analyzed by deproteinizing the plasma with ethanol and drying the sample by rotary evaporation. KIC was then derivatized with *o*-phenylenediamine - extracted in ethanol and dried again. The dried KIC is derivatized using MTBSTFA and m/z of 259 and 260; it is analyzed with GCMS. Amino acid concentrations in plasma were analyzed by high pressure liquid chromatography (HPLC) using on-column derivatization with ortho-phtaldialdehyde/3-mercaptopropionic acid (OPA/3-MPA) as described earlier [13]. Breath samples were analyzed for ^13^CO_2_ using a BreathMat (Thermo Finnegan, Bremen, Germany).

Whole-body rate of appearances (Ra) for phenylalanine and leucine were calculated:

$$\mathrm{Ra}=I/\left(\left(E_{i}/E_{A}\right)-1\right),$$

in which I is the infusion rate of the tracer and E_i_ and E_A_ are the enrichments in the infusate and plasma respectively. Plasma KIC enrichments were used for leucine Ra calculation, and Ra plasma enrichments of ^2^H_5_-pheylalanine and ^2^H_2_-tyrosine were used for phenylalanine and tyrosine, respectively. The endogenous rate of appearance (endoRa) was calculated by subtracting the amino acid infusions in the parenteral nutrition from the Ra. Both amino acids are essential, and therefore the endoRa represents the whole-body protein breakdown rate.

Because these measurements were done in a steady-state situation, the Ra is equal to the rate of disappearance (Rd). Whole body protein synthesis was calculated by subtracting the oxidation rates for phenylalanine and leucine from the Rd. Oxidation rates for leucine (Ox) were estimated by the appearance of ^13^CO_2_ in the breath and for phenylalanine by the flux to tyrosine (Q_PT_). The plasma KIC enrichments were used for the leucine calculations because they better represent the intracellular events [14]:

$$\begin{array}{l} Q_{\mathrm{PT}}={\mathrm{Ra}}_{\mathrm{Tyr}}\times \left(E_{\mathrm{ATyr}}/E_{\mathrm{APhe}}\right)\times {\mathrm{Ra}}_{\mathrm{Phe}}/\left(I_{\mathrm{Phe}}+{\mathrm{Ra}}_{\mathrm{Phe}}\right)\\ \mathrm{Leu}\;\mathrm{Ox}=0.8\left(V_{\mathrm{CO}2}\times^{13}{\mathrm{CO}}_{2\mathrm{breath}}\right)/E_{\mathrm{KIC}} \end{array}$$

in which E_ATyr_ and E_APhe_ are the plasma enrichments of ^2^H_4−_tyrosine and ^2^H_5_-phenylalanine, I_Phe_ is the infusion rate of the phenylalanine tracer, V_CO2_ is the CO_2_ production rate as measured by the DeltaTrac Metabolic Monitor, ^13^CO_2_ is the enrichment in the breath samples and E_KIC_ is the enrichment of ^13^C-KIC in the plasma. The factor 0.8 is to correct for the recovery of ^13^CO_2_ during the study period [15].

Whole-body protein synthesis rates were estimated by subtracting the Ox from the Rd. Whole-body protein balance was calculated by subtracting protein breakdown (Ra phenylalanine or leucine) from this protein synthesis rate.

Student’s *t*-test for paired samples was used for comparing the measurements in the hypocaloric and normocaloric states. The Kolmogorov-Smirnov test was used to test for normal distribution of the data. When applicable (as indicated in the text), Wilcoxon’s rank test was applied. A *P*-value <0.05 in a two-sided test was considered statistically significant. STATISTICA software (Statsoft, Uppsala, Sweden) was used for calculations. The sample size of 16 subjects was chosen to enable us to combine the two randomized groups into one by only applying a simple non-parametric sign test (8 subjects in each group). In the literature the coefficient of variation for whole-body leucine kinetics in healthy subjects is reported to 3 to 4% [16].

## Results

**Figure 2:**
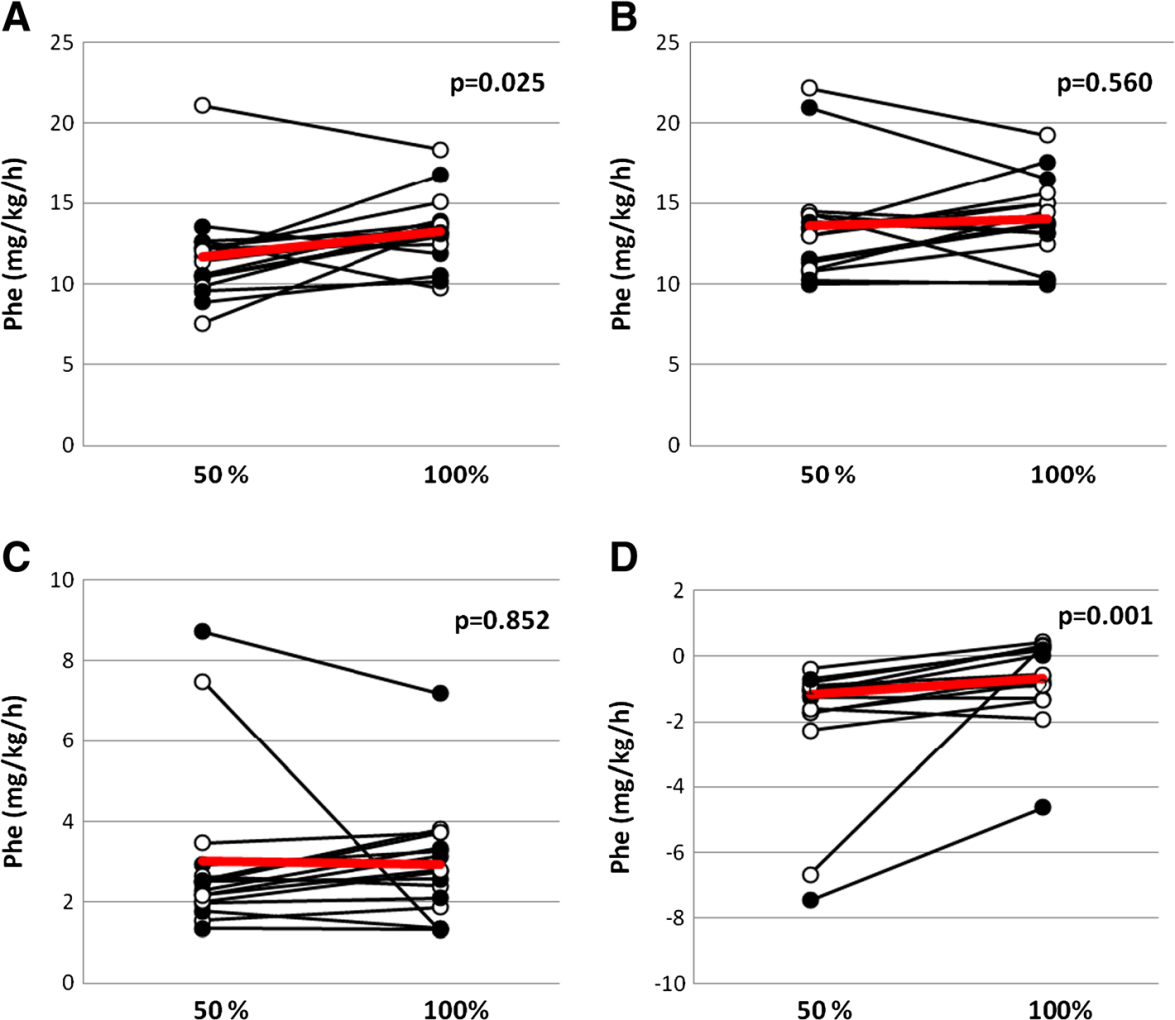
**Whole body protein kinetics, calculated using a phenylalanine tracer, in critically ill mechanically ventilated neurosurgical patients (n = 16) who received normocaloric and hypocaloric intravenous nutrition in random order during two consecutive days.** Patients were randomized to receive normocaloric before hypocaloric (filled symbols), or hypocaloric before normocaloric (open symbols) nutrition. **(A)** Whole-body protein synthesis; **(B)** whole-body protein degradation; **(C)** phenylalanine oxidation; **(D)** whole-body protein balance. *P*-values are for paired comparisons. Phe, phenylalanine. Red lines indicate mean values.

Patients (n = 16) with traumatic brain injury or intracranial bleeding were studied. They were sedated, and were on mechanical ventilation and parenteral nutrition. Seventeen patients were recruited into the study, but one patient had to be excluded due to protocol violation in terms of non-adhesion to the nutritional protocol. The mean (± SD) age of the patients was 49.5 ± 10.4 years. The male/female gender distribution was 11/5 (see Table 1 for other patient characteristics). After energy expenditure measurement by indirect calorimetry, patients were randomized into the two groups to be studied for 48 hours, namely, hypocaloric before normocaloric, or normocaloric before hypocaloric. Patients were studied on day 6 (range 1 to 17 days) of the ICU stay; when given normocaloric feeding the total parenteral nutrition (TPN) contained 21.7 ± 4.1 kcal/kg/24 h and 1.07 ± 0.15 g protein/kg/24 h (Table 1). For individual patients, part of the energy supply was contained in the sedation given. The two patient subgroups were compared statistically for all variables shown below; no significant differences were observed. In Figures 1 and 2 the two subgroups are indicated.

**Table 1:** Patient characteristics

**Weight (kg)**	**Diagnosis**	**GCS, admission (score)**	**Study day (in ICU)**	**SOFA (study day)**	**Survival**	**Group**^**a**^	**EE (kcal/kg)**	**TPN (kcal/kg)**	**Protein (g/kg)**
Individual patients									
79	TBI	3	17	5	Y	100/50	24.9	18.8	0.9
95	SDH	12	4	9	N	50/100	23.5	23.5	1.1
86	SDH	12	4	12	Y	100/50	25.8	24.6	1.2
76	SDH	3	8	9	Y	50/100	22.4	18.4	0.9
85	TBI	6	3	10	Y	100/50	27.0	25.9	1.3
77	ICH	6	10	6	Y	50/100	23.0	19.1	0.9
61	SAH	11	17	7	Y	100/50	31.3	20.3	1.2
78	TBI	6	5	9	Y	50/100	24.1	24.1	1.2
90	SAH	5	5	9	N	100/50	20.6	17.0	0.8
64	SDH	5	5	10	Y	50/100	25.8	16.4	0.8
65	EDH	4	4	9	Y	100/50	26.4	26.4	1.3
87	SDH	3	3	3	Y	50/100	19.9	19.9	1.0
89	SDH	4	1	7	Y	50/100	28.8	28.8	1.4
95	TBI	3	5	9	Y	100/50	27.1	26.5	1.3
100	SDH	5	11	7	Y	50/100	25.7	22.5	1.1
85	SDH	8	1	9	Y	100/50	18.9	15.5	0.8
Full patient sample (n = 16), mean values (SD)									
82.0	NA	6.0	6.4	8.1	NA	NA	24.7	21.7	1.1
(11.5)	NA	(3.1)	(5.0)	(2.2)	NA	NA	(3.3)	(4.1)	(0.2)

^a^Percent measured energy expenditure (EE) during 24 hours/percent measured EE during the next consecutive 24 hours. TBI, traumatic brain injury; SDH, subdural hemorrhage; ICH, ischemic brain injury; SAH, subarachnoid hemorrhage; EDH, epidural hemorrhage; GCS, Glasgow coma scale; SOFA, sequential organ failure assessment score; EE, energy expenditure; TPN, total parenteral nutrition; Y, yes; N, no; NA, not applicable.

Indirect calorimetry was repeated three times for each patient; (i) before randomization, (ii) after 24 hours of being given parenteral nutrition (PN) of 100% of measured EE, and (iii) after 24 hours of being given PN oft 50% of measured EN (Figure 1). Pre-study EE was 24.7 ± 3.3 kcal/kg/24 h, which was not different from the energy expenditure obtained after being given 24 hours of PN at 100% of measured EE, 24.5 ± 2.3 kcal/kg/24 h, while the measured energy expenditure after being given 24 hours of 50% of measured EE was 4% lower at 23.4 ± 2.4 kcal/kg/24 h (*P* = 0.049). For the respiratory quotient, the pre-study value was not different from the value obtained after being given 24 hours of PN at 100% of measured EE, 0.80 ± 0.10 and 0.82 ± 0.10, respectively. On the other hand, after being given 24 hours of PN of 50% of measured EE the respiratory quotient was lower at 0.76 ± 0.01 (*P* = 0.005) compared to the value obtained after 24 hours of 100% of measured EE.

Because the amount of protein given was different due to the use of an all-in-one formulation, the free amino acids were measured in plasma. The total sum of free amino acids in plasma did not change significantly, with 2.8 ± 0.5 mmol/L versus 2.9 ± 0.4 mmol/L (*P* = 0.085) for PN of 50% and 100% of measured EE, respectively.

Insulin administration at the time of protein turnover measurements was recorded retrospectively. Eight patients were not given insulin; the remaining eight patients were given 5.8 ± 5.0 (median 4.0) U/h when given 100% of EE, and 3.2 ± 2.1 (median 3.5) U/h when 50% of EE was given. When all 16 patients are combined the corresponding values are 2.9 ± 3.8 (median 1.0) U/h and 1.6 ± 2.7 (median 0.0) U/h (*P* >0.05 (Wilcoxon test) in both calculations).

Whole-body protein turnover was measured during the last 30 minutes of 24 hours of PN, corresponding to 50% or 100% of measured EE (see Figure 2 for measurements with a phenylalanine tracer). Whole-body protein synthesis was 12% lower when 50% of EE was given, 11.7 ± 3.0 versus 13.3 ± 2.2 mg phenylalanine/kg/h (*P* = 0.025), whereas whole-body protein degradation was also unaltered at 13.6 ± 3.5 versus 14.0 ± 2.6 mg phenylalanine/kg/h (*P* = 0.56). Whole-body protein oxidation was unaltered at 3.0 ± 2.1 versus 2.9 ± 1.4 mg phenylalanine/kg/h (*P* = 0.85), but the difference in the whole-body synthesis rate resulted in a 60% difference in whole-body protein balance, with −1.9 ± 2.1 versus −0.7 ± 1.3 mg phenylalanine/kg/h (*P* = 0.001, Wilcoxon test).

**Figure 3:**
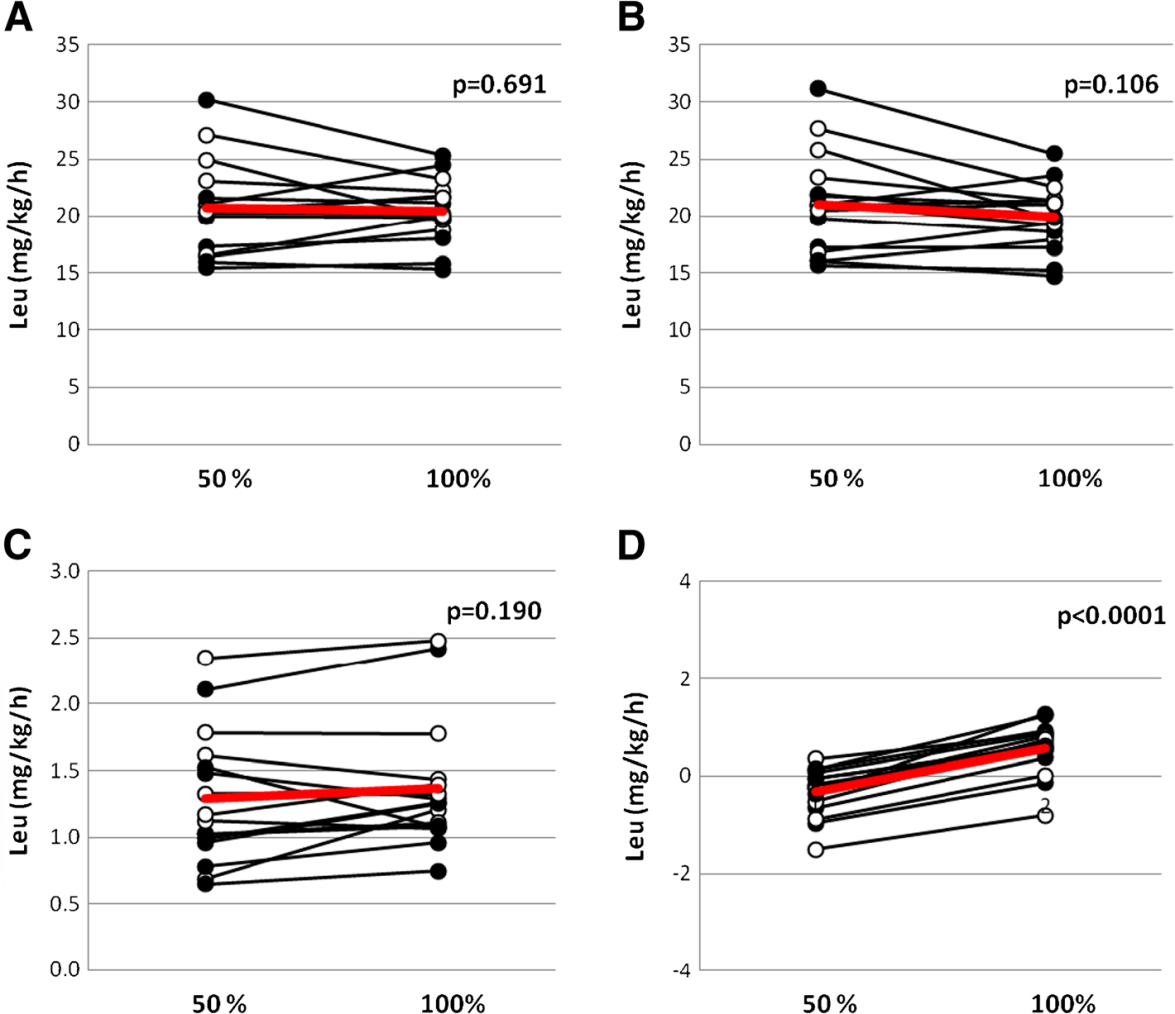
**Whole body protein kinetics, calculated using a leucine tracer, in critically ill mechanically ventilated neurosurgical patients (n = 16) who received normocaloric and hypocaloric intravenous nutrition in random order during two consecutive days.** Patients were randomized to receive normocaloric before hypocaloric (filled symbols), or hypocaloric before normocaloric (open symbols) nutrition. **(A)** Whole-body protein synthesis; **(B)** whole-body protein degradation; **(C)** leucine oxidation; and **(D)** whole-body protein balance. *P*-values are for paired comparisons. Leu, leucine. Red lines indicate mean values.

Figure 3 illustrates the results with a leucine tracer. The difference in whole-body protein balance remained at −0.3 ± 0.5 compared to 0.6 ± 0.5 mg leucine/kg/h (*P* <0.001) for PN of 50% and 100% of measured EE, respectively, and simultaneously the whole-body protein oxidation was unaltered at 1.3 ± 0.5 versus 1.4 ± 0.5 mg leucine/kg/h (*P* = 0.19). For whole-body synthesis and degradation the results were different; whole-body protein synthesis was unaltered at 20.7 ± 4.1 versus 20.5 ± 2.8 mg leucine/kg/h (*P* = 0.69), and so was whole-body protein degradation at 21.0 ± 4.4 versus 19.9 ± 2.8 mg leucine/kg/h (*P* = 0.11).

## Discussion

Whole-body protein kinetics were studied in patients in the neurosurgical ICU, who were sedated and on mechanical ventilation, and were receiving hypocaloric and normocaloric feeding by standard PN. The major finding was that full feeding resulted in a less negative or positive whole-body protein balance, and that simultaneously the amino acid oxidation was not different.

The study protocol using two consecutive days, during which patients received two different treatments, is an attractive option in studies of heterogenous patient groups, such as critically ill patients [17-19]. Due to randomization of the time order of treatments and comparability of patients' characteristics on the two days, we can assume that conditions outside the intervention were reasonably similar and therefore, that this enabled a fair comparison. The study of neurosurgical patients on mechanical ventilation also made indirect calorimetry applicable, as the oxygen fraction was low in all patients.

The study protocol focused on optimizing measurement conditions by means of (i) a homogenous group of patients, (ii) high probability of two comparable consecutive 24-hour periods, and (iii) full control over nutrition by the parenteral route. Naturally, these inclusion criteria limit the external validity of the study, but under these conditions, the nutrition made a difference for whole-body protein kinetics, which was not just a combusted caloric intake, as reflected by the amino acid oxidation. It is, however, beyond the scope of the study to speculate over the possible clinical relevance of this finding. Still, it is reasonable to conclude that the results demonstrate the usefulness of the applied technique and protocol, which suggests its use in studies in the future.

Measured EE determined the caloric supply, whereas the composition of the specific all-in-one formulation used determined the protein supply. The protein supply level was below the recommendation given in the European Society of Clinical Nutrition and Metabolism (ESPEN) guidelines [1]. An interesting observation is that the amino acid oxidation level was not different between the two levels of protein supply, which suggests that a protein supply of 1.07 + 0.15 g protein/kg/24 h was effectively utilized. Future studies will address the question of the optimal protein supply level [20].

In an earlier study including singular measurements in a small group of general ICU patients and of volunteers, we also used phenylalanine and leucine tracers in parallel [21]. The rationale for using parallel tracers was that existing literature on whole-body protein kinetics evaluated by these two tracers, tends to show a uniform result for the protein balance level, but the contributions from changes in whole-body protein synthesis and breakdown may be related to the amino acid tracer used [22]. Our pilot experience reproduced that finding. So, in the present study we continued to use parallel tracers and again found this difference. The phenylalanine tracer indicated a less negative whole-body protein balance when receiving 100% of EE related to a higher rate of whole-body protein synthesis, while the leucine tracer gave a similar result for whole-body protein balance, related to a tendency (not statistically significant) towards a lower rate of protein degradation.

The mechanism(s) behind the varying results obtained with the phenylalanine and leucine tracers is/are obscure. A possible explanation is the variation in representation in the precursor pools of individual organs by the two tracers. Phenylalanine probably better represents the liver, while leucine (or more correctly α-keto-iso-caproate, which is used for the calculations) better represents skeletal muscle.

The generalizability of our data is limited due to: (i) the study population having narrow inclusion criteria, (ii) patients being on PN only, and (iii) the low nitrogen-to-calorie ratio in the commercially available formulation that was used. So, the difference between hypocaloric and normocaloric supply may be attributed to the administered caloric levels or to the protein (amino acid) levels, or a combination of the two. Clearly, further studies are needed to settle this point. The time course during the ICU stay, and the possibility of an adaptation period after changes in protein intake, are other issues that need to be addressed in the future.

The limitation related to the use of PN only constitutes a technical problem that should not be underestimated in critically ill patients, where the success rate of enteral feeding related to the prescribed dose or to nutritional target is most often low and unpredictable [23]. In the present and future studies, selection of patients on PN only, or patients on successful and stable enteral feeding, also will considerably limit external validity. Techniques and protocols that are generalizable to most critically ill patients being fed enterally, parenterally, or by a combination of the two, must be developed.

In the present study involving neurolosurgical ICU patients the observed EE level ranged from 19 to 31 kcal/kg/24 h (median 25) during the initial week of the ICU stay. In a general ICU population the values are often lower during the initial week of stay (range 14 to 34 kcal/kg/24 h; median 21) as shown in a recent study [24]. It may be that the patient group in this study had a certain level of hypermetabolism (not severe), but we cannot confirm this as a factor that invalidates our results. Hypermetabolism is repeatedly reported in studies of neurosurgical critically ill patients [25]. The small but statistically significant difference in measured EE between pre-study and 50% PN measurements may be interpreted as a sign of overfeeding before the study period, at least in some cases. The possibility of an insulin effect related to the higher caloric intake during the normocaloric feeding period was also considered. However, no such effect was detected.

## Conclusion

In this pilot, whole-body protein kinetics were investigated in a selected group of critically ill patients in the neurosurgical ICU. Hypocaloric feeding was found to be associated with a more negative protein balance, but with unaltered amino acid oxidation, compared to normocaloric feeding. Overall the protein content of the feeding given was low, 0.53 g protein/kg/24 h and 1.07 g protein/kg/24 h, respectively, in the two feeding protocols. The major finding of the study, which evaluated the nutritional support effect on critically ill patients, is the usefulness of the whole-body protein turnover technique, combined with a 48-hour protocol, when comparing two alternative feeding protocols in the individual patient.

## Key messages

• Whole-body protein turnover measurement is a useful technique to elucidate the protein content of feeding in the critically ill.

• Full nutrition with a standard IV formula gives a better protein balance compared to hypocaloric feeding.

• A protein supply of 1.1 g/kg/day as part of full feeding does not enhance amino acid oxidation, which would have been indicative of futile protein supply.

## Abbreviations

endoRa: Endogenous rate of appearance; ESPEN: European Society of Clinical Nutrition and Metabolism; GCMS: Gas chromatography mass spectrometry; HPLC: High pressure liquid chromatography; IC: Indirect calorimetry; IV: Intravenous; kcal: Kilocalorie; KIC: Keto-iso-caproate; Ox: Oxidation rate; PN: Parenteral nutrition; Ra: Rate of appearance; Rd: Rate of disappearance; TPN: Total parenteral nutrition.

## Competing interests

None of the authors have any competing interest to declare.

## Authors’ contributions

AB, OR, and JW: conception and design of the study, interpretation of data, finalizing manuscript. AB and BMB: acquisition of data. OR: analytical procedures and methods. AB, OR and JW: calculations and manuscript preparation. All authors read and approved the final manuscript.

## Authors’ information

Agneta Berg, RNA PhD: Postdoctoral Fellow at the Division of Anaesthesiology at CLINTEC, Karolinska Institutet, and Head Nurse at Department of Neonatology Karolinska University Hospital, Huddinge; at the time of the study, nurse at the Department of Neurosurgery, Karolinska University Hospital, Solna.

Olav Rooyackers, PhD: Professor of Anesthesiology and Intensive Care Medicine at the Division of Anaesthesiology at CLINTEC, Karolinska Institutet.

Bo-Michael Bellander, MD, PhD: Associate Professor of Neurosurgery at the Division of Neurosurgery at Clinical Neuroscience, Karolinska Institutet, and senior consultant at the Department of Neurosurgery, Karolinska University Hospital, Solna.

Jan Wernerman, MD, PhD: Professor of Anesthesiology and Intensive Care Medicine at the Division of Anaesthesiology at CLINTEC, Karolinska Institutet, and senior consultant at the Department of Anesthesiology and Intensive Care Medicine, Karolinska University Hospital Huddinge.
